# Undescended Testes Growth Potential in Relation to Testis Position from Diagnosis until Puberty

**DOI:** 10.3390/jcm13092620

**Published:** 2024-04-29

**Authors:** Maciej Nowak, Jerzy Niedzielski, Jolanta Slowikowska-Hilczer, Renata Walczak-Jedrzejowska, Katarzyna Marchlewska

**Affiliations:** 1 Department of Pediatric Surgery and Urology, University Pediatric Centre, Central University Hospital, Medical University of Lodz, 90-419 Lodz, Poland; maciej-roman.nowak@umed.lodz.pl (M.N.); jerzy.niedzielski@umed.lodz.pl (J.N.); 2 Department of Andrology and Reproductive Endocrinology, Central University Hospital, Medical University of Lodz, 90-419 Lodz, Poland; jolanta.slowikowska-hilczer@umed.lodz.pl (J.S.-H.); renata.walczak-jedrzejowska@umed.lodz.pl (R.W.-J.)

**Keywords:** undescended testis, intraabdominal testis, testicular volume, testicular atrophy index, testicular growth potential

## Abstract

**Background**: Testicular volume (TV) and testicular atrophy index (TAI) were used to determine criteria for normal, hypotrophic and atrophic undescended testes (UDT). **Objectives**: This study aimed to determine changes in TV and TAI in patients with different types of UDT. **Materials and Methods**: 182 boys (aged 0.3–14.0 years) with 212 UDTs were assessed twice 24 months apart. Testes were unilateral (UCT) or bilateral canalicular (BCT) and intra-abdominal (IAT). **Results**: At the beginning of the observation, the highest TAI was observed in IAT and the lowest in the BCT group (38.1 vs. 12.5%, *p* < 0.05). After 2 years, the highest TAI was observed in the BCT and IAT groups (20.5 and 19.1%), while the lowest was in the UCT group (12.0%, *p* < 0.05). At the beginning and after 2 years, the highest TAI was observed in boys aged < 6 years (25.0%, 18.2%) and the lowest in pubertal boys aged 12–14 years (5.9%, 7.3%, *p* < 0.05). A total of 78.3% of patients at the beginning and 86.8% at the end of the observation had TAI < 30%. Furthermore, 7% of boys at the beginning and 3% at the end of the observation had TAI > 50%. IATs have the highest testicular growth potential (TGP), while BCTs have the lowest (120.0 vs. 28.6%, *p* < 0.05). The highest TGP was in boys aged < 3 years (100%, *p* < 0.05) and boys aged 12–14 years (98.1%, *p* < 0.05), while the lowest was in boys aged 9–10.9 years (19.5%, *p* < 0.05). **Conclusions:** We revealed the continuous growth of UDTs until puberty independently of their position. IATs revealed high growth potential.

## 1. Introduction

An undescended testis (UDT) may be situated in any position along its normal route of descent. Most UDTs are canalicular (palpable) and only about 6% are non-palpable [1]. Non-palpable UDT means that the testis is either intra-abdominal (IAT) or absent (agenesis or vanishing testis) [1,2].

The assessment of testicle size in the scrotum can be subjective (orchidometer) or objective (ultrasonography—US). However, UDT, palpable or not, can be measured in the US only, and its volume (TV), calculated based on its three dimensions, is an important predicting factor for future testis function [3]. A significant decrease in TV in UDT present at a high location and in patients treated later during childhood was reported by several authors [2,4,5,6].

Although TV provides valuable information about the testis, its value must be related to the norms in a specific age group. However, it does not allow for a comparison between age groups, and most importantly, changes in the TV of the gonad appearing with the patient’s age do not allow for a reliable interpretation of results.

All these comparisons are available with the testicular atrophy index (TAI), which the authors have been using for over three decades [7]. TAI shows as a percentage how much the TV of the affected testicle differs from the TV of the healthy one. TAI enables a reliable comparative analysis in a given patient in different age periods, allows comparing patients in different age groups, and determines the criterion of values for hypotrophic and atrophic testes.

The aim of this study was to determine the values of TV and TAI, their changes with advancing age, and mutual correlations in patients with UDT divided into three groups: unilateral canalicular, bilateral canalicular, and intra-abdominal testes.

The authors’ idea was to conduct the study in a narrow time frame at two time points 24 months apart in patients with different types of UDT, who were at different ages and at different stages of development at the time of examination.

## 2. Materials and Methods

Included in the study were 182 boys (aged 0.3–14.0 years) with congenital UDT visiting the Surgical Clinic of the Ambulatory Care Unit of the University Pediatric Centre, Central University Hospital of Medical University in Lodz, Poland, between May 2020 and April 2023.

Approval for the study was obtained from the Bioethics Committee of the Medical University of Lodz (No RNN/43/15/KE of 17 March 2015).

### 2.1. Patients

Patients were operated on between 2009 and 2021. Surgery was performed at 8 months to 2 years of age in boys with intra-abdominal testes and at 10 months to 9 years of age in boys with unilateral and bilateral canalicular undescended testes. No patient received hormonal therapy.

In each patient, the position and size of the gonad were assessed twice: at the beginning and the end of the study approximately 24 months apart. In some cases, the first examination was performed before surgery and the second one after. In others, both examinations were performed after surgery. 

All the patients were otherwise healthy. No patient was excluded from the study.

The position and the three dimensions of both testes were recorded by means of ultrasonography and used to calculate TV: TV (cm^3^) = 0.52 × width (cm) × length (cm) × height (cm). TAI of the affected testicle was calculated: TAI (%) = [(contralateral TV − affected TV)/contralateral TV] × 100% [7]. The TAI of the bilateral UDTs was calculated in reference to the median TV of healthy gonads of unilateral canalicular UDT boys of the appropriate age group. Testes with TAI > 30% were considered as hypotrophic and those with TAI > 50% as atrophic. We also calculated the testicular growth percentage (TGP) as the percentage difference in TV measured at the beginning and the end of the observation: TGP (%) = [(TVend − TVbeg)/TVbeg] × 100%.

The patients were divided into age subgroups based on the developmental changes in testes: 0–2.9, 3–5.9, 6–8.9, 9–10.9, 11–12 (TV < 4 mL, non-pubertal boys—np) and 12–14 (TV ≥ 4 mL, pubertal boys—p) years [8]. Participants’ ages were expressed in decimals to avoid presentation in months. The age value was expressed as the quotient of the number of months for a given child divided by 12. Another division was based on the type of disorder: unilateral canalicular (UCT), bilateral canalicular (BCT) and intra-abdominal (IAT) UDT (Table 1).

### 2.2. Surgical Procedures

A total of 182 patients with 212 UDTs were operated on (Table 1). Boys with palpable testes underwent an open surgery via an inguinal approach; the testis’ position and size were examined. Orchidofunicolysis and orchidopexy were performed by the conventional technique of placing the testis in the subdartos pouch [1,6]. Boys with bilateral palpable UDT underwent inguinal orchidopexy first of the bigger or lower testis and of the other testis 3 to 6 months later.

As an initial procedure, boys with impalpable testes underwent diagnostic laparoscopy with a Fowler–Stephens (FS) operation for IAT. Next, the inguinal orchidopexy was performed 6–9 months later [1]. Boys with bilateral IAT underwent a simultaneous bilateral laparoscopic F-S procedure, followed by inguinal orchidopexy first of the bigger or lower testis, and the other testis 3 to 6 months later.

### 2.3. Statistics

All the analyses were performed using Statistica 13.1 for Windows (StatSoft Inc., Tulsa, OK, USA). The distribution of the data was analyzed using the Shapiro–Wilk test. The data were distributed in a nonparametric manner, so, they were presented as median and range and analyzed using the ANOVA Kruskal–Wallis test for the assessment of the statistical difference between the groups. Spearman’s rank correlation (r_s_—coefficient) was conducted between the results of the examined parameters. The Student *t*-test and Pearson’s or Fisher’s exact chi-squared test were used for a comparison of the examined parameter values and the incidence in the studied groups of patients. Differences were considered significant at *p* < 0.05.

## 3. Results

### 3.1. Entire Group

Direct comparisons of the healthy and undescended testes’ size between the groups were not performed as these would have been directly dependent upon the patients’ age. The significantly lower median TV of UDT was observed in boys aged < 9 years with IAT at the beginning and end of the observation in comparison to boys with UCT and BCT. TV of UDT did not differ significantly in UCT and BCT groups independently of patients’ age.

**Figure 1:**
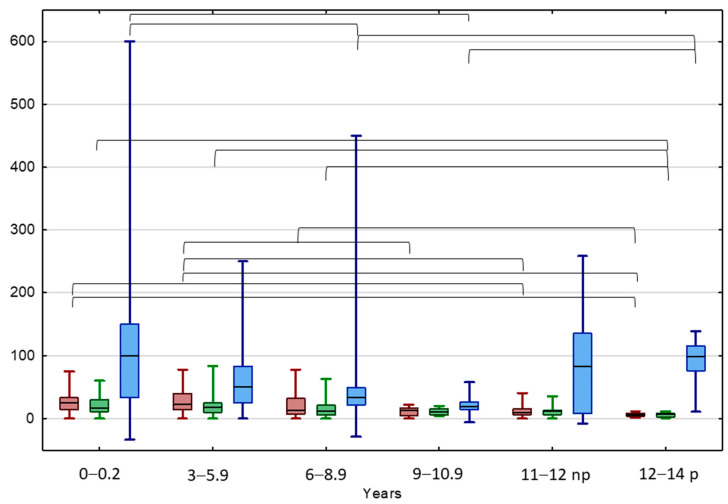
Comparison between TAI before (red) and after treatment (green) and TGP (blue) in different age groups of patients with UDTs. Results are presented as median (line), lower and upper quartiles (box) and minimum–maximum (whisker); np—non-pubertal boys at age 11–12 y., TV < 4 mL; p—pubertal boys at age 12–14 y. TV ≥ 4 mL. Statistically significant differences are presented by the black horizontal brackets. ANOVA Kruskal–Wallis test, *p* < 0.05.

A better tool for the estimation of testicular volume changes seems the TAI value, indicating the percentage loss of the affected testicle volume in relation to the healthy testicle, regardless of the absolute volume of the testicle related to the patient’s age. At the beginning of the observation, the highest median TAI value was observed in the IAT group and the lowest in the BCT group (38.1 vs. 12.5%, *p* < 0.05). However, at the end of the observation, the highest TAI values were observed in the BCT and IAT groups (20.5 and 19.1%, respectively), while the lowest in the UCT group (12.0%, *p* < 0.05). At the beginning of the observation, the highest median TAI value was observed in boys aged < 6 years (25.0%, 22.2%) and the lowest in pubertal boys aged 12–14 years (5.9%, *p* < 0.05). At the end of the observation, the highest TAI value was in boys aged < 6 years (16.7%, 18.2%), while the lowest was again in pubertal boys aged 12–14 years (7.3%, *p* < 0.05) (Figure 1).

In the entire study group, 78.3% of UDTs had TAI < 30%, which means that both testes had normal TV at the beginning of the study. This percentage increased significantly after 2 years of observation to 86.8%. The highest number of normal testes, both at the beginning and at the end of the study, was observed in the UCT boys. The smallest numbers of normal testicles both at the beginning and at the end of the study were found in IAT boys (Table 2).

In the entire study group, 7% of UDTs had a TAI exceeding 50% at the beginning of the study. This percentage decreased significantly after 2 years of observation to about 3%. The smallest number of hypotrophic testicles both at the beginning and the end of the study was observed in the boys with UCT. The highest number of atrophic testes was observed in the IAT boys at the beginning of the study; however, no atrophic testes were found in this group of patients at the end of the observation (Table 2).

**Figure 2:**
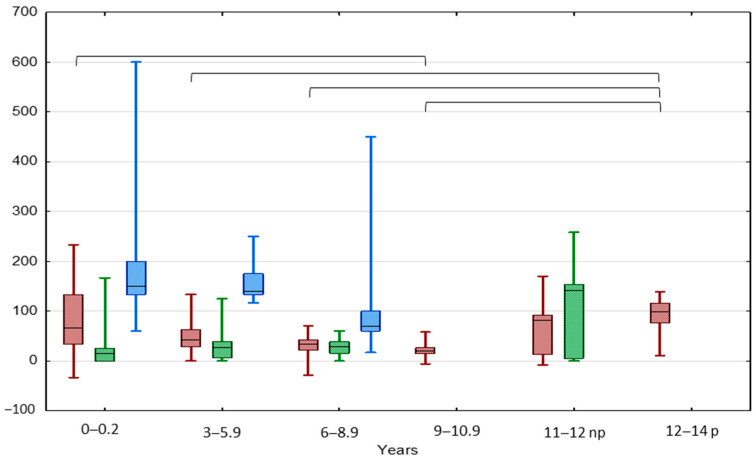
Comparison between TGP in patients with UCT (red), BCT (green), and IAT (blue) in different age groups. Results are presented as median (line), lower and upper quartiles (box) and minimum–maximum (whisker); np—non-pubertal boys at age 11–12 y., TV < 4 mL; p—pubertal boys at age 12–14 y. TV ≥ 4 mL. Statistically significant differences are presented by the black horizontal brackets. ANOVA Kruskal–Wallis test, *p* < 0.05.

The highest median TGP value of UDT was In the IAT group and the lowest was in the BCT group (120.0 vs. 28.6%, *p* < 0.05) (Figure 1 and Figure 2). The highest median TGP value of UDT was in boys aged < 3 years (100%, *p* < 0.05) and pubertal boys aged 12–14 years (98.1%, *p* < 0.05), while the lowest was in boys aged 9–10.5 years (19.5%, *p* < 0.05).

A significant negative correlation was found between the TGP values and the patient’s age (r_s_ −0.23; *p* = 0.0008) (Supplementary Materials: Table S1).

### 3.2. UCT Group

**Figure 3:**
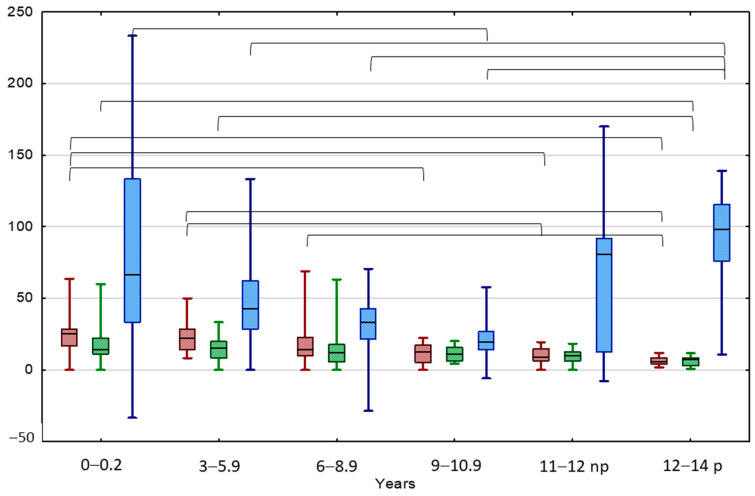
Comparison between TAI before (red) and after treatment (green), and TGP (blue) in different age groups of patients with UCT. Results are presented as median (line), lower and upper quartiles (box) and minimum–maximum (whisker); np—non-pubertal boys at age 11–12 y., TV < 4 mL; p—pubertal boys at age 12–14 y. TV ≥ 4 mL. Statistically significant differences are presented by the black horizontal brackets. ANOVA Kruskal–Wallis test, *p* < 0.05.

TV of the normal testes significantly increased with boys’ age, both at the beginning and at the end of the observation (Table 3, Figure 3). By analogy, the TV value of UDT increased significantly with boys’ age, both in the beginning and at the end of the observation. A significant positive correlation between TV values at the beginning of the observation and after 24 months was found in the entire UCT group, as well as in each age subgroup (Supplementary Materials: Table S1). 

TAI decreased with boys’ age, both at the beginning and at the end of the observation (Table 3). The TAI value in boys aged < 9 years was significantly higher at the beginning of the observation in comparison to patients aged 12–14 years old, starting puberty. The TAI value in boys aged < 6 years was significantly higher after 24 months of observation than the TAI values of patients aged 12–14 years old. A significant positive correlation between TAI values at the beginning and the end of the observation was found in the entire UCT group and boys aged 6–8.9 years and >11 years, non-pubertal and pubertal. A significant negative correlation was found between the patient’s age and TAI values both at the beginning and the end of the observation.

The TGP of normal testis was 50% in boys aged < 3 years, then decreased to about 20% between 3 and 11 years, then increased significantly up to about 80% in non-pubertal boys aged 11–12 years old and 100% in pubertal boys (Table 3, Figure 3). The UDTs behaved similarly: their TGP value was 66.7% in boys aged < 3 years; then, this significantly decreased to 19.5% in boys of 9–10.9 years, then increased significantly up to 98.1% in pubertal boys aged 12–14 years old. An interesting significant positive correlation between the TAI and the TGP values at the beginning of the observation was observed in the youngest boys aged < 6 years, while after treatment these parameters correlated significantly negatively in the groups aged < 3 and 9–10.5 years.

### 3.3. BCT Group

The TV value of UDT both at the beginning and the end of the observation correlated significantly positively with the boys’ age, but a significant increase was observed only in non-pubertal boys aged 11–12 years in comparison to other age groups. The TV values of UDT in each age subgroup in both observational points were lower in comparison to the average for healthy testes calculated, respectively, in each age subgroup of UCT (Table 3). A positive correlation between TV values at the beginning of the observation and after about 2 years was found in the entire BCT group as well as in each age subgroup, except for boys aged < 3 years (Supplementary Materials: Table S1).

**Figure 4:**
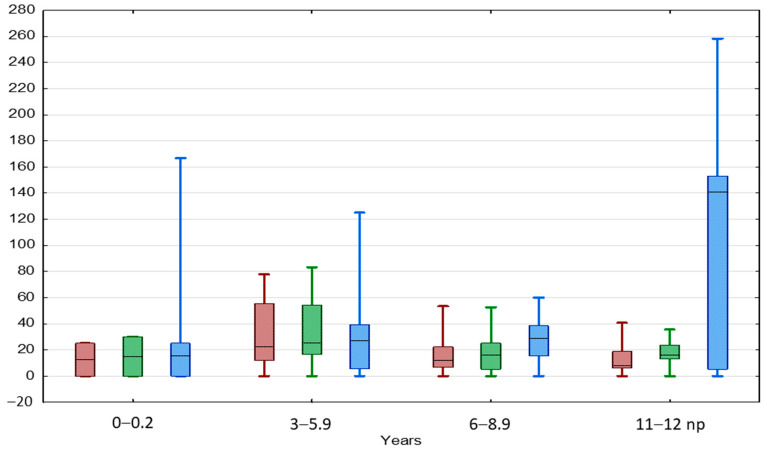
Comparison between TAI before (red) and after treatment (green) and TGP (blue) in different age groups of patients with BCT. Results are presented as median (line), lower and upper quartiles (box) and minimum–maximum (whisker). ANOVA Kruskal–Wallis test, *p* < 0.05.

The TAI values showed a slow insignificant downward trend with boys’ age keeping values below 25%, both in the beginning and at the end of the observation (Table 3, Figure 4). A positive significant correlation between TAI values in the beginning and at the end of the observation was found in the entire BCT group as well as in each age subgroup, except for boys aged < 3 and 11–12 years. 

The TGP showed a slow insignificant upward trend with boys’ age to the level of 28.6% in boys aged 6–8.9 years, then increased significantly up to 140.6% in boys aged 11–12 years (Table 3).

### 3.4. IAT Group

The TV values of normal testicles increased significantly with boys’ age, both at the beginning and at the end of the observation (Table 3). By analogy, the TV values of UDTs increased significantly with boys’ age, both at the beginning and at the end of the observation (Table 3). A significant positive correlation between TV values at the beginning of the observation and after 24 months was found in the entire IAT group and boys under 6 years old.

**Figure 5:**
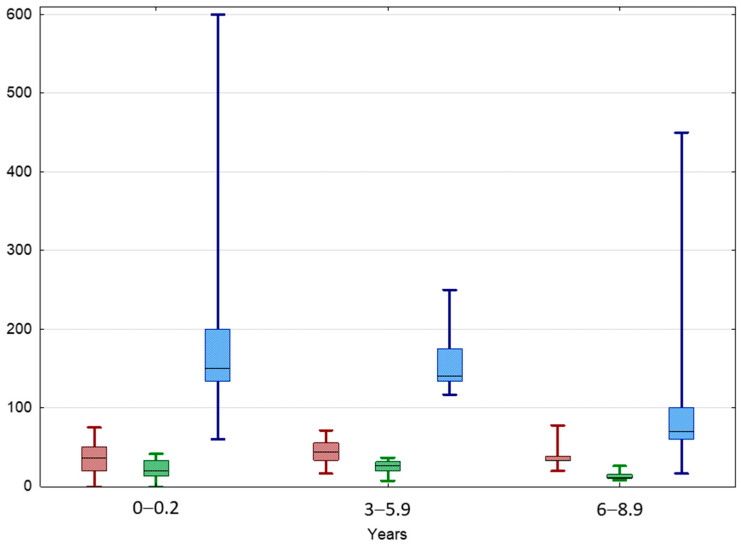
Comparison between TAI before (red) and after treatment (green) and TGP (blue) in different age groups of patients with IAT. Results are presented as median (line), lower and upper quartiles (box) and minimum–maximum (whisker). ANOVA Kruskal–Wallis test, *p* < 0.05.

The TAI value increased slightly in boys aged 3–6 years, then decreased with boys’ age, both in the beginning and at the end of the observation (Table 3, Figure 5). A significant positive correlation between TAI values in the beginning and at the end of the observation was found in the entire IAT group and boys aged < 6 years (Supplementary Materials: Table S1).

The TGP of normal testes was 100% in boys aged < 3 years and 111% in boys aged 3–5.9 years, then significantly dropped to 26.7% in boys aged 6–8.9 years (Table 3, Figure 5). UDTs behaved similarly; their TGP value was 150% in boys aged < 3 years and 140% in boys aged 3–5.9 years, then dropped to 70% in boys aged 6–8.9 years. A significant positive correlation between TGP and TAI values at the beginning of the observation was found in the entire IAT group as well as in each age subgroup.

## 4. Discussion

The prepubertal testis after the minipuberty period of life is frequently considered not active hormonally; spermatogenesis is arrested at the spermatogonia level, and the volume of the testes does not change before the onset of puberty. However, it was found that although the size of the gonads increases almost threefold during childhood, the change in absolute volume is small and, thus, clinically undetectable [8,9,10]. It was found that testicular volume increases due to the growth in length of seminiferous tubules, which results from the active proliferation of infantile Sertoli cells [8]. Immature Sertoli cells are hormonally active and secrete estradiol and anti-Müllerian hormone (AMH), increase FSH receptors, and respond to FSH. Spermatogonia proliferate by mitosis but do not enter meiosis. In our study, we confirmed that normal descended testes consistently increased their volume during the prepubertal and early pubertal periods of life.

UDTs frequently are reported as gonads with a significantly smaller volume in comparison with normal testes and decrease with advancing age, which is more evident in testes present at high locations and patients treated later during childhood [11,12,13,14]. However, in our study in the UCT group, UDT showed a similar growth rate to normal testes with the advancing age, both at the beginning and at the end of the observation. The higher was age the lowest was TAI in the beginning and at the end of the observation, which means that, unilaterally, UDT has high potential to develop. The TAI value was significantly lower in pubertal boys at the beginning and after 2 years of observation in comparison to younger patients. The TGP of UDT was high (median: 67%) in the youngest group aged < 3 years, but surprisingly, it was the highest in pubertal boys (median 98–100%). To our knowledge, this is the first description of the same growth potential of unilateral UDTs and the healthy testicles in boys during puberty. Tseng et al. [13] observed that orchidopexy performed within one year from birth significantly accelerated the growth of a unilateral UDT compared to a group aged > 2 years (mean: 3.9), which confirms the suggestion that orchidopexy should be performed before one year of age. However, our results revealed that unilateral UDT may have normal potential for growth even during puberty.

A different situation was found in the group of boys with BCT where the TV value of UDT both at the beginning and the end of the observation correlated significantly positively with age only in boys aged > 6 years in comparison to other age groups. The TV of UDT in each age group was lower than the median among healthy testicles; however, it was significantly higher in comparison to TV in the IAT group. In this group, TAI did not decrease significantly, even at the beginning of puberty, but remained below 25% compared to the average of healthy testes. In the BCT group, there was no correlation between age and TAI or TGP. TGP was the highest in prepubertal boys aged 11–12 years old, which may suggest that testicles in older boys have greater growth potential. BCTs grow slowly in their rhythm, regardless of the boy’s age. They have much lower growth dynamics compared to UCT boys, which may confirm suggestions that they may be more seriously damaged [15,16]. Later in time, males with bilateral undescended testes show lower fertility rates than men with unilateral disease [17,18].

In our study, we observed the lowest volume and the highest TAI of testes in the IAT group, which confirms the observations of other authors [19,20]. Unexpectedly, significant growth of IAT occurred in all age groups. In our previous study, we revealed that the higher the UDT position, the more frequently (and more) defects accompanied it. We found also that the higher the gonad position, the more severe the observed atrophy of the testes. However, in the present study, we observed that although testes initially located in the abdominal cavity have a smaller volume compared to the canalicular testes during the prepubertal period of life, they still show a gradual increase in volume. Interestingly, TGP after 2 years of observation was the highest in the group of boys with IAT in comparison to other groups. Moreover, TAI > 50% (atrophic testes) after 2 years was not revealed, although in younger boys, it was the highest among studied groups. IATs are considered the most damaged, usually dysgenetic, and have little chance for future fertility [21,22,23]. However, Aijiki [24] suggested that post-orchidopexy testicular atrophy may occur regardless of the patient’s age at orchidopexy, and orchidopexy is recommended irrespective of age at diagnosis. We revealed that despite these observations, IATs may have the potential to grow. Other authors [25,26] found that TV was correlated with the age-related number of germ cells (gonocytes, spermatogonia, and spermatocytes) per cross-sectional seminiferous tubule in adult patients and was prognostic for later fertility. Thus, the increasing volume of IAT may show developing spermatogenesis, but one would need a further follow-up of the studied boys to confirm this.

In our study, the GTP value was the highest in the youngest children. In the boys with IAT, the younger the patient was, the faster the growth of the testes, which confirms the general recommendations to treat UDT as early as possible, especially those in the abdominal position. Tseng et al. [27] observed that an undescended testicle grew faster when orchiopexy was performed before one year of age. However, in older boys, GTP was still significantly higher in IAT than in boys with UCT and BCT. TAI was significantly higher in all age groups with IAT in the beginning of the observation and in boys aged < 6 years at the end in comparison to other groups. However, in boys aged 6–8.9 years old, TAI was the lowest. Taskinen and Wikstrom [5] measured TV in 75 adults treated for cryptorchidism when they were 10 months to 13 years old. The authors suggested that early orchidopexy at an age younger than 2 years is not necessarily essential, because the preoperative location of the testis in otherwise-healthy boys exerts no definite effect on the final testicular volume. However, adult TV was slightly greater in patients with cryptorchidism if treated at ages up to 5 years. Also, Sijstermans et al. [11] suggest that orchidopexy for congenital UDT, even performed later than current recommendations [28,29], does not result in severe testicular growth retardation. In turn, Hildorf [30] found that even though cryptorchid boys undergo orchidopexy within the first year of age, up to 25% may risk later infertility.

The limitation of this study is the low number of patients with different ages and UDT positions, especially in the BCT and IAT groups, where pubertal patients were absent. However, preliminary conclusions could be drawn. In turn, the strength of the study is the presence of patients above 3 years of age with UDT observed after orchidopexy.

## 5. Conclusions

We revealed the following: (1) the continuous growth of undescended testes until puberty independently of their position; (2) the high growth potential of intraabdominal testes, although they were smaller than unilateral and bilateral canalicular UDTs.

## Figures and Tables

**Table 1:** Patients’ characteristics.

	Unilateral Canalicular UDT	Bilateral Canalicular UDT	Intra-Abdominal UDT	Total
Age				
Years-range	0.3–14	1.2–12	0.9–9.1	0.3–14
Mean ± SD	6.6 ± 4.3	6.5 ± 3.6	3.2 ± 4.2	6.0 ± 4.0
Median	5.4	6.3	2.5	4.7
Age groups	[n (%)]	[n (%)]	[n (%)]	[n (%)]
0–2.9	37 (28.9)	5 (21.7)	21 (67.7)	63 (34.6)
3–5.9	29 (22.7)	6 (26.1)	6 (19.3)	41 (22.5)
6–8.9	17 (13.3)	7 (30.4)	4 (12.9)	28 (15.4)
9–10.9	10 (7.8)	-	-	10 (5.5)
11–12 np	20 (15.6)	5 (21.7)	-	25 (13.7)
12–14 p	15 (11.7)	-	-	15 (8.2)
Total				
Patients [n (%)]	128 (70.3)	23 (12.6)	31 (17.0)	182 (100)
Testes [n (%)]	128 (60.4)	46 (21.7)	38 (17.9)	212 (100)
Testis location	[n (%)]	[n (%)]	[n (%)]	[n (%)]
R	73 (57.0)	-	11 (28.9)	84 (39.6)
L	55 (43.0)	-	11 (28.9)	66 (31.1)
Bil	-	46 (100)	16 (42.1)	62 (29.2)

Abbreviations: Bil—bilateral, L—left, np—non-pubertal, p—pubertal, R—right, UDT—undescended testis.

**Table 2:** TAI values in consecutive clinical groups at the beginning and the end of the observation.

TAI		<30%	31–50%	>50%	Total
UCT [n (%)]	Beginning	116 (90.6)	9 (9.0)	3 (2.3)	128
	End	120 (93.7)	6 (4.7)	2 (1.6)	128
P		NS	NS	NS	
BCT [n (%)]	Beginning	39 (84.8)	4 (8.7)	3 (6.5)	46
	End	38 (82.6)	4 (8.7)	4 (8.7)	46
P		NS	NS	NS	
IAT [n (%)]	Beginning	11 (28.9)	18 (47.4)	9 (40.0)	38
	End	26 (66.4)	12 (31.6)	0 (0.0)	38
P		**0.028**	NS	**0.026**	
Total [n (%)]	Beginning	166 (78.3)	31 (14.6)	15 (7.1)	212
	End	184 (86.8)	22 (10.4)	6 (2.8)	212
P		**0.039**	NS	NS	

NS—non-significant; BCT—bilateral canalicular UDT, IAT—intra-abdominal testes, TAI—testicular atrophy index, UCT—unilateral canalicular UDT, Chi-square test; in bold: *p* < 0.05.

**Table 3:** TV (mL), TAI (%) and TGP (%) values of healthy gonads and UDTs in consecutive clinical and age subgroups at the beginning (B) and end (E) of the study.

Age (years)	Parameters	UCT		BCT		IAT	
		N	Median (Min–Max)	N	Median (Min–Max)	N	Median (Min–Max)
0–2.9	TV healthy (B)	37	0.6 (0.2–1.2)	37	0.6	24	0.4 (0.3–0.8)
0–2.9	TV healthy (E)		0.9 (0.3–1.3)		0.9		0.9 (0.6–1.3)
0–2.9	TV UDT (B)	37	0.4 (0.1–0.9)	10	0.7 (0.3–0.8)	24	0.3 (0.1–0.5)
0–2.9	TV UDT (E)		0.7 (0.2–1.0)		0.8 (0.7–1.0)		0.7 (0.4–1.2)
0–2.9	TAI (B)		25.0 (0–63.6)		12.5 (0–25.0)		36.7 (0–75.0)
0–2.9	TAI (E)		14.3 (0–60.0)		15.0 (0–30.0)		20 (0–41.7)
0–2.9	TGP healthy		50.0 (0.0–200.0)		-		100 (50–200)
0–2.9	TGP UDT		66.7 (−33.3–233.3)		15.5 (0–166.7)		150 (60.0–600.0)
3–5.9	TV healthy (B)	29	0.9 (0.6–1.2)	29	0.9	9	0.9 (0.6–1.0)
3–5.9	TV healthy (E)		1.2 (0.8–2.0)		1.2		1.8 (1.1–1.9)
3–5.9	TV UDT (B)	29	0.7 (0.4–1.1)	12	0.7 (0.2–0.9)	9	0.5 (0.2–0.7)
3–5.9	TV UDT (E)		1.0 (0.7–1.7)		0.9 (0.2–1.1)		1.2 (0.7–1.6)
3–5.9	TAI (B)		22.2 (8.3–50.0)		22.2 (0–77.8)		44.4 (16.7–71.4)
3–5.9	TAI (E)		15.0 (0–33.3)		25.0 (0–83.3)		26.3 (7.7–36.8)
3–5.9	TGP healthy		22.2 (9.1–128.6)		-		111.1 (55.6–116.7)
3–5.9	TGP UDT		42.9 (0.0–133.3)		26.8 (0–125.0)		140 (116.7–250.0)
6–8.9	TV healthy (B)	17	1.4 (0.8–2.2)	17	1.4	5	1.5 (1.3–1.8)
6–8.9	TV healthy (E)		1.7 (0.8–3.5)		1.7		1.9 (1.8–2.4)
6–8.9	TV UDT (B)	17	1.2 (0.5–1.7)	14	1.3 (0.5–1.4)	5	1.0 (0.4–1.2)
6–8.9	TV UDT (E)		1.5 (0.5–2.9)		1.5 (0.8–2.0)		1.6 (1.4–2.2)
6–8.9	TAI (B)		14.3 (0.0–68.8)		12.2 (0–53.3)		33.3 (20.0–77.8)
6–8.9	TAI (E)		12.0 (0.0–63.2)		15.8 (0–52.6)		11.1 (8.3–26.3)
6–8.9	TGP healthy		20.0 (0.0–78.6)		-		26.7 (26.7–38.5)
6–8.9	TGP UDT		33.3 (−28.6–70.6)		28.6 (0–60.0)		70.0 (16.7–450.0)
9–10.9	TV healthy (B)	10	1.8 (1.4–2.3)	-	-	-	-
9–10.9	TV healthy (E)		2.2 (1.8–3.2)		-		-
9–10.9	TV UDT (B)	10	1.7 (1.4–1.9)		-		-
9–10.9	TV UDT (E)		2.0 (1.6–3.0)		-		-
9–10.9	TAI (B)		12.7 (0.0–22.2)		-		-
9–10.9	TAI (E)		10.8 (4.3–20.0)		-		-
9–10.9	TGP healthy		19.3 (4.5–39.1)		-		-
9–10.9	TGP UDT		19.5 (−5.9–57.9)		-		-
11–12 np	TV healthy (B)	20	3.3 (2.1–3.9)	20	3.3	-	-
11–12 np	TV healthy (E)		5.6 (2.7–7.0)		5.6		-
11–12 np	TV UDT (B)	20	3.0 (1.7–3.6)	10	3.0 (1.9–3.6)		-
11–12 np	TV UDT (E)		5.2 (2.4–6.4)		7.0 (2.0–12.9)		-
11–12 np	TAI (B)		9.1 (0–19.4)		7.8 (0–40.6)		-
11–12 np	TAI (E)		9.7 (0–18.4)		16.1 (0–35.5)		-
11–12 np	TGP healthy		79.7 (2.6–154.5)		-		-
11–12 np	TGP UDT		80.7 (−7.7–170.0)		140.6 (0–258.3)		-
12–14 p	TV healthy (B)	15	5.3 (4.0–6.8)	-	-		-
12–14 p	TV healthy (E)		10.7 (4.5–13.7)		-		-
12–14 p	TV UDT (B)	15	5.2 (3.6–6.4)		-		-
12–14 p	TV UDT (E)		10.6 (4.1–12.9)		-		-
12–14 p	TAI (B)		5.8 (1.8–11.9)		-		-
12–14 p	TAI (E)		7.4 (0.9–11.5)		-		-
12–14 p	TGP healthy		100.0 (7.1–134.5)		-		-
12–14 p	TGP UDT		98.1 (10.8–138.9)		-		-

Abbreviations: BCT—bilateral canalicular UDT, IAT—intra-abdominal testes, TAI—testicular atrophy index, TGP—testicular growth percentage, TV—testicular volume, UCT—unilateral canalicular UDT, UDT—undescended testis.

## Data Availability

The data presented in this study are available on reasonable request from the corresponding author.

## Supplementary Materials

The following supporting information can be downloaded at: https://www.mdpi.com/article/10.3390/jcm13092620/s1, Table S1: Spearman rank correlation.
