# Supplementary material for: Undescended Testes Growth Potential in Relation to Testis Position from Diagnosis until Puberty
Source: J Clin Med. 2024 Apr 29;13(9):2620. doi: 10.3390/jcm13092620 (PMC11084872; doi:10.3390/jcm13092620)
Supplement: Supplementary file 1 [file jcm-13-02620-s001.zip › jcm-2962864-supplementary.pdf]

Table S1. Spearman rank correlation. The marked correlation coefficients are significant with p <0.05000

Parameter	Age	TV healthy (B)	TV healthy (E)	TV UDT (B)	TV UDT (E)	TAI (B)	TAI (E)	TGP UDT
**The entire study group**								
Age		0,951	0,916	0,893	0,867	-0,453	-0,261	-0,229
TV healthy (B)	0,951		0,937	0,925	0,879	-0,436	-0,251	-0,275
TV healthy (E)	0,916	0,937		0,854	0,932	-0,404	-0,230	-0,053
TV UDT (B)	0,893	0,925	0,854		0,897	-0,683	-0,406	-0,359
TV UDT (E)	0,867	0,879	0,932	0,897		-0,565	-0,482	-0,016
TAI (B)	-0,453	-0,436	-0,404	-0,683	-0,565		0,593	0,289
TAI (E)	-0,261	-0,251	-0,230	-0,406	-0,482	0,593		-0,080
TGP UDT	-0,229	-0,275	-0,053	-0,359	-0,016	0,289	-0,080	
**Unilateral canalicular UDT**								
Age		0,954	0,914	0,937	0,892	-0,582	-0,351	-0,080
TV healthy (B)	0,954		0,953	0,966	0,917	-0,561	-0,333	-0,100
TV healthy (E)	0,914	0,953		0,929	0,970	-0,567	-0,335	0,092
TV UDT (B)	0,937	0,966	0,929		0,940	-0,693	-0,409	-0,131
TV UDT (E)	0,892	0,917	0,970	0,940		-0,635	-0,477	0,123
TAI (B)	-0,582	-0,561	-0,567	-0,693	-0,635		0,536	0,137
TAI (E)	-0,351	-0,333	-0,335	-0,409	-0,477	0,536		-0,218
TGP UDT	-0,080	-0,100	0,092	-0,131	0,123	0,137	-0,218	
**Bilateral canalicular UDT**								
Age		0,972	0,976	0,801	0,817	-0,279	-0,061	0,250
TV healthy (B)	0,972		0,996	0,802	0,807	-0,237	-0,024	0,185
TV healthy (E)	0,976	0,996		0,805	0,812	-0,262	-0,023	0,229
TV UDT (B)	0,801	0,802	0,805		0,940	-0,715	-0,407	0,154
TV UDT (E)	0,817	0,807	0,812	0,940		-0,604	-0,527	0,394
TAI (B)	-0,279	-0,237	-0,262	-0,715	-0,604		0,604	-0,085
TAI (E)	-0,061	-0,024	-0,023	-0,407	-0,527	0,604		-0,399
TGP UDT	0,250	0,185	0,229	0,154	0,394	-0,085	-0,399	
**Intra-abdominal UDT**								
Age		0,919	0,917	0,805	0,880	0,071	-0,152	-0,281
TV healthy (B)	0,919		0,922	0,761	0,827	0,249	-0,017	-0,293
TV healthy (E)	0,917	0,922		0,771	0,902	0,140	-0,039	-0,186
TV UDT (B)	0,805	0,761	0,771		0,899	-0,411	-0,430	-0,667
TV UDT (E)	0,880	0,827	0,902	0,899		-0,180	-0,430	-0,324
TAI (B)	0,071	0,249	0,140	-0,411	-0,180		0,628	0,588
TAI (E)	-0,152	-0,017	-0,039	-0,430	-0,430	0,628		0,275
TGP UDT	-0,281	-0,293	-0,186	-0,667	-0,324	0,588	0,275	

Abbreviations: UDT- undescended testis, TAI - testicular atrophy index, TGP – testicular growth percentage, TV – testicular volume, (B) - beginning, (E) – end.
